# PI-103 and Quercetin Attenuate PI3K-AKT Signaling Pathway in T- Cell Lymphoma Exposed to Hydrogen Peroxide

**DOI:** 10.1371/journal.pone.0160686

**Published:** 2016-08-05

**Authors:** Akhilendra Kumar Maurya, Manjula Vinayak

**Affiliations:** Biochemistry & Molecular Biology Laboratory, Centre for Advanced Study in Zoology, Institute of Science, Banaras Hindu University, Varanasi-221005, India; Suzhou University, CHINA

## Abstract

Phosphatidylinositol 3 kinase—protein kinase B (PI3K-AKT) pathway has been considered as major drug target site due to its frequent activation in cancer. AKT regulates the activity of various targets to promote tumorigenesis and metastasis. Accumulation of reactive oxygen species (ROS) has been linked to oxidative stress and regulation of signaling pathways for metabolic adaptation of tumor microenvironment. Hydrogen peroxide (H_2_O_2_) in this context is used as ROS source for oxidative stress preconditioning. Antioxidants are commonly considered to be beneficial to reduce detrimental effects of ROS and are recommended as dietary supplements. Quercetin, a ubiquitous bioactive flavonoid is a dietary component which has attracted much of interest due to its potential health-promoting effects. Present study is aimed to analyze PI3K-AKT signaling pathway in H_2_O_2_ exposed Dalton’s lymphoma ascite (DLA) cells. Further, regulation of PI3K-AKT pathway by quercetin as well as PI-103, an inhibitor of PI3K was analyzed. Exposure of H_2_O_2_ (1mM H_2_O_2_ for 30min) to DLA cells caused ROS accumulation and resulted in increased phosphorylation of PI3K and downstream proteins PDK1 and AKT (Ser-473 and Thr-308), cell survival factors BAD and ERK1/2, as well as TNFR1. However, level of tumor suppressor PTEN was declined. Both PI-103 & quercetin suppressed the enhanced level of ROS and significantly down-regulated phosphorylation of AKT, PDK1, BAD and level of TNFR1 as well as increased the level of PTEN in H_2_O_2_ induced lymphoma cells. The overall result suggests that quercetin and PI3K inhibitor PI-103 attenuate PI3K-AKT pathway in a similar mechanism.

## Introduction

PI3K is crucial signal transducing enzyme regulating cell proliferation, cell survival, differentiation, apoptosis and angiogenesis [1, 2]. It is essential for activation of AKT which plays a central role in both physiological and pathological signaling mechanisms. PI3K-AKT pathway is major drug target due to its frequent activation in cancer [3–9]. PI3K is a lipid kinase, responsible for phosphorylation of PIP2 to PIP3 which is the activation site for AKT (or protein kinase B/PKB) and PDK. PI3K family is divided into three classes (class I, II & III) which differ in structure, substrate preference, tissue distribution, mechanism of activation and in function [10–12]. Class I PI3K has a long history of association with cancer. It is a heterodimer composed of a catalytic subunit P110α and regulatory subunit p85α [11–15]. PI3K dependant AKT activation leads to multistep process involving both membrane translocation and phosphorylation [16]. AKT is phosphorylated at Thr-308 in kinase activation loop and Ser-473 at carboxyl terminal. Thr-308 is phosphorylated by PDK1 whereas PDK2 is responsible for phosphorylation of Ser-473 [17, 18]. PDK1 is a crucial kinase required for normal mammalian development [19, 20]. AKT is comprised of 3 isoforms: AKT1, AKT2 and AKT3 according to different tissue distribution and biological activities. AKT1 plays a major role in regulation of cell survival and angiogenesis [3, 11, 21].

Cell survival is promoted by AKT mediated phosphorylation and inhibition of pro-apoptotic protein BAD [22]. BAD is a member of Bcl-2 family that promotes cell death by displacing Bax from binding to Bcl-2 and Bcl-xL [23]. Inactivation of BAD is also mediated through phosphorylation by ERK activated p90 ribosomal S6 kinase [24]. ERK is widely expressed signaling molecule that participates in regulation of a large variety of processes including cell adhesion, cell cycle progression, cell migration, cell survival, differentiation, metabolismand proliferation [24]. PI3K activation is responsible for ERK1/2 phosphorylation [25]. PKCα-mediated activation of ERK1/2 has also been reported through MEK [26].

Highly reactive oxygen species like hydrogen peroxide (H_2_O_2_), superoxide anion (O2^•−^), hydroxyl radicals (OH^.^) etc., produced in cells are grouped as reactive oxygen species (ROS) which confer reactivity to different biological targets. It has been suggested that ROS is selected by nature for adaptation to changes in environmental nutrients and oxidative environment during evolution [27]. Disturbance in balance between production of ROS and organism’s antioxidant defence system leads to accumulation of ROS causing oxidative stress. Oxidative stress is closely related to all aspects of cancer. It has been linked to hyper-activation of signaling pathways and metabolic adaptations of tumor microenvironment. Sustained oxidative stress in tumor microenvironment is due to production of ROS by tumor cells themselves and by activated neutrophils and macrophages. Recent reports indicate that apart from gene mutation by oxidative damage of cellular macromolecules including DNA, ROS has direct or indirect role in modulation of signal transduction and transcription factors to regulate cell survival, proliferation and migration [27–31]. Oxidative stress has been linked to hyper-activation of signaling pathways and metabolic adaptations of tumor microenvironment.

Induction of lymphoma cells by external exposure of H_2_O_2_ is hypothesized to be counterbalanced by an antioxidant supplement. Being flavonoid, quercetin (QUE) is an antioxidant rich in onion, grape, red wine etc. It has attracted much of interest due to its potential health-promoting effects against cardiovascular diseases, diabetes, thrombosis, longevity, including prevention against certain forms of cancer [32]. Being a dietary product, QUE is less likely to induce normal tissue toxicity and is reported to have no adverse health effects [11, 32, 33].

H_2_O_2_ is most commonly used as source of ROS for oxidative stress preconditioning [29, 34]. It is formed by dismutation of superoxide (O2•−) spontaneously or enzymatically, catalyzed by superoxide dismutase (SOD). H_2_O_2_ is more stable as compared to other ROS having half life of 1ms, and serves as inter as well as intracellular signaling molecule as it easily crosses membrane and diffuses from site of production [35, 36]. Aquaporin 8 (AQP8) in the inner mitochondrial membrane has a role in transport of H_2_O_2_ [33].

PI3K is activated by several factors including ROS [37]. ROS mediated signaling leading to activation of PI3K-AKT pathway plays an important role in the development of cancer. Estrogen induced ROS is reported to cause increased phosphorylation of AKT in breast cancer. Recruitment of ROS is required for PI3K-AKT activation in T-cell acute lymphoblastic leukemia cells **[38].** Tumor microenvironment not only plays a pivotal role during cancer progression and metastasis but also has profound effects on therapeutic efficacy. AKT is an important driver of the tumor glycolytic phenotype and stimulates ATP generation. Increased intracellular glucose level is necessaryfor enhanced glycolytic metabolism. Lactate dehydrogenase (LDH)-A is a glycolytic enzyme that favours the conversion of pyruvate to lactate. It is important for maintenance and progression of tumor growth as oxygen availability is limiting in tumor microenvironment. Elevated LDH-A is important for tumor initiation to meet energy demand. High LDH-A in DL mice has been previously reported by our group which has been suppressed by various natural antioxidants **[39]**. We have earlier reported decreased activity of LDH-A by QUE which is correlated with markedly delayed tumor formation in DL mice **[11].** AKT stimulates glycolysis by increasing the expression and membrane translocation of glucose transporters and by phosphorylating key glycolytic enzymes such as hexokinase and phosphofructokinase2 as well as phosphorylating mTOR **[40]**. Activated mTOR stimulates protein and lipid biosynthesis and cell growth in response to sufficient nutrient and energy conditions during tumorigenesis. PI-103 is a potent, cell permeable, inhibitor of catalytic subunit of PI3K (P110α) which competes with ATP binding site. It represents an exploratory compound for investigating the therapeutic relevance of PI3K inhibitors in cancer [41]. PI-103 inhibits proliferation and invasion of a wide variety of cancer cells and shows corresponding modulation of various cancer biomarkers [41, 42].

The present study is aimed to analyze the molecular mechanism of PI3K dependent modulation in H_2_O_2_ induced DLA cells. Further, the impact of QUE is compared with PI-103 to regulate H_2_O_2_ induced PI3K-AKT pathway.

## Materials and Methods

### Chemicals

All chemicals used were of molecular biology and analytical grade. Quercetin, Dichlorofluorescein diacetate (H_2_DCFDA), horseradish peroxidise (HRP) conjugated β-actin, anti-rabbit phospho p85α were purchased from Sigma Aldrich (St. Louis, MO); PI-103 from Cayman (Ann Arbor, MI); anti-rabbit AKT1, anti-rabbit phospho AKT Ser-473, anti-rabbit phospho AKT Thr-308, anti-rabbit p85α, anti-rabbit PTEN, anti-rabbit phospho BAD, anti-rabbit phospho PDK1 from Cell Signaling Technology (Danvers, MA); anti-rabbit ERK1/2, anti-rabbit phospho ERK1/2, anti-rabbit TNFR1 from Biovision (Milpitas, CA); anti-rabbit PKCα from Santa Cruz Biotechnology (Dallas, Texas), HRP conjugated goat anti-rabbit secondary antibody from Bangalore Genei (Bangalore, India); enhanced chemiluminescence (ECL) Super Signal Kit from Pierce Biotechnology (Rockford, IL) and H_2_O_2_ from S D Fine Chem Limited (Mumbai, India).

### Cell culture and treatment

Dalton’s lymphoma ascite (DLA) cells were grown *in vitro* from ascite cells collected from DL bearing mice. AKR strain mice were bred and maintained under standard laboratory conditions with proper human care, as per the guidelines of Institutional Animal Ethical Committee, Banaras Hindu University, Varanasi, at 25±2°C under a 12h light/12h dark schedule with standard mice feed and drinking water *ad libitum*. All the experiments were approved by Central Animal Ethical Committee, Banaras Hindu University (Letter no. Dean/10-11/92). Healthy adult male mice (16–20 weeks old and 30±2g) were used in experimental work. About 1x10^6^ viable ascite cells in 1mL of PBS per mouse were transplanted to adult male mice intraperitoneally (i.p.) as described earlier [11, 43].

DL mice were sacrificed by humanely euthanization (cervical dislocation after anesthetized; mice were exposed to ether presented on a cotton gauze inside a small chamber and ether concentration was approximately 80μL/L of volume of the container). Under aseptic condition, DLA cells were grown and maintained in RPMI (Roswell Park Memorial Institute) medium supplemented with 10% FBS (fetal bovine serum), 2mM L-glutamine, 100IU/mL penicillin and 100μg/mL streptomycin. Cells were incubated in CO_2_ incubator (Sanyo) with a humidified atmosphere and 5% CO_2_ at 37°C.

Dose and time for H_2_O_2_ treatment—1mM for 30min was based on literature survey [34] as well as pilot study performed in our lab. Approximately, 5 X 10^5^DLA cells/mL containing culture medium on 6 well plates was exposed to H_2_O_2_ and further proceeds for biochemical and molecular analysis. In another experiment, DLA cells were pre-treated with PI-103 (10μM) or QUE (300μM) for 6h and treated with H_2_O_2_.

### Total ROS measurements

Total ROS level was determined by oxidative conversion of nonfluorescent 2’, 7’-dichlorofluorescein diacetate (H_2_DCFDA) to highly fluorescent 2’, 7’-dichlorofluorescein (DCF) as described previously [44]. Cell extracts of amount 100μl were incubated at 37°C for 60min with 2mM of H_2_DCFDA (sigma) in PBS. Fluorescence was recorded at 485nm (excitation) and 527nm (emission) with fluorescence spectrophotometer (HITACHI F-3000). The level of ROS in each sample was determined by observing fluorescence (absorbance)/mg protein.

### Flow cytometric analysis of ROS

Cellular ROS contents were measured by flow cytometry. Briefly, 5 X 10^5^cells were seeded on 6 well plates and exposed to H_2_O_2_ at 1mM for 30min. Further, cells were washed with PBS and stained with 10μM of H_2_DCFDA at 37°C for 30min. Cells were collected and fluorescence was analyzed using a FACS Calibur flow cytometer (BD Bioscience, CA). Data were analyzed by CellQuest^TM^ pro-software (BD Bioscience, CA).

### Cytotoxicity by MTT Assay

DLA cell cytotoxicity was analysed as described previously [45], approximately 30 x 10^3^ cells were seeded in each well of a 96 well microtiter plates in 100 μL complete culture media containing PI-103 (10 μM) and QUE (300 μM) for 6 h. Thereafter, cells were treated with H_2_O_2_ for 30 min. Equivalent concentration of DMSO (vehicle) was added to control wells. After incubation, 100 μL of MTT solution (stock 5 mg/ml in PBS) was added into each well for 3 h. Pellet was dissolved in 100 μL of DMSO and the absorption of formazan solution was measured at 570 nm using a micro plate reader (ECIL).

### Western blotting

Cells were lysed in buffer containing 20mM Tris-HCl (pH 7.4), 150mM NaCl, 1mM EDTA, 1mM EGTA, 1% Triton X-100 and 1mM PMSF as described previously [43]. Cellular debris was spun down at 14,000g for 20min at 4°C and supernatant was used as whole protein extract. Isolated protein was quantified using Bradford reagent. Equal amount of protein from each sample was separated using 10% SDS-PAGE and transferred to a PVDF membrane overnight at 4°C. Membrane was blocked in 5% non-fat milk in PBS (pH 7.4) for 2h at RT. Further, Membrane was probed separately with primary antibodies anti-rabbit phospho p85α (1:500 dilution), anti-rabbit AKT1 (1:1000 dilution), anti-rabbit phospho AKT Ser-473 (1:1000 dilution), anti-rabbit phospho AKT Thr-308 (1:1000 dilution), anti-rabbit p85α (1:1000 dilution), anti-rabbit PTEN (1:1000 dilution), anti-rabbit PKCα (1:500 dilution), anti-rabbit phospho BAD (1:1000 dilution), anti-rabbit phospho PDK1 (1:1000 dilution), anti-rabbit ERK1/2 (1:500 dilution), anti-rabbit phospho ERK1/2 (1:500 dilution), anti-rabbit TNFR1 in 1% BSA and 0.05% Tween-20 in PBS (PBST; pH 7.4) overnight at 4°C. After thorough washing in 1X PBS for 3min, blot was incubated with HRP-conjugated goat anti-rabbit immunoglobulin G (IgG) (1:2500 dilution; Bangalore Genei, Bangalore, India) in PBST (pH 7.4) containing 5% non-fat milk and 0.05% Tween-20 for 2h at RT. Immunoreactive protein was detected using ECL Super Signal Kit (Pierce Biotechnology) on X-ray film. Intensity of bands was analyzed by densitometric scanning using Gel Doc System (Alpha Innotech^EC^). Relative densitometric values were calculated after normalization with β-actin.

### Statistical analysis

All experiments were repeated three times independently and one representative image is presented in the figures. Student’s t-test was used for statistical analysis between control and H_2_O_2_-treated groups. One-way analysis of variance (ANOVA) followed by Tukey test were used for statistical analysis to compare the significant difference between control, H_2_O_2_-treated group, and H_2_O_2_-treated with PI-103 or QUE treated group. Data represent as mean ± S.E.M. * (#) denotes significant differences at level of p < 0.05. # indicates significant difference between control *vs* H_2_O_2_-treated group; and * H_2_O_2_ treated group *vs* H_2_O_2_ with PI-103 or QUE treated group.

## Results

Cytotoxicity of 1 mM of H_2_O_2_ was checked on DLA cells and no significant difference in cell viability was found.

### H_2_O_2_ induces ROS accumulation

**Fig 1:**
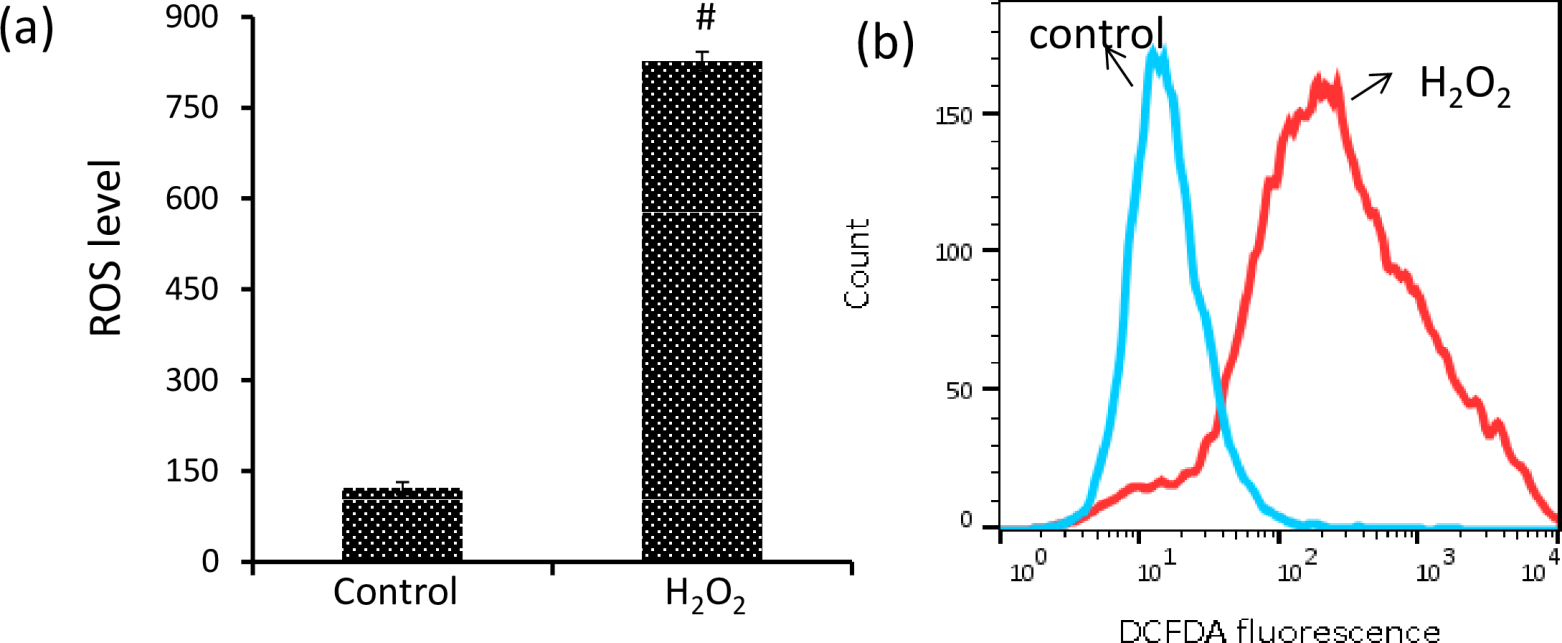
H_2_O_2_ induces ROS accumulation. (a) Homogenates of DLA cells from 3 set of each group were pooled separately and used for total ROS measurement (b) Cells were loaded with H_2_DCFDA (10μM) for 30min and amount of ROS generated was indicated by shift in fluorescence as detected by flow cytometry. Student’s t-test was used for statistical analysis. Data represent as mean ± S.E.M. of three independent experiments. * denotes significant differences at the level of p < 0.05 between H_2_O_2_-treated and control group.

The effect of H_2_O_2_ was analyzed on ROS level of DLA cells in terms of absorbance (fluorescence/mg protein) using green fluorescent dye, H_2_DCFDA. ROS level was increased significantly by approximately 5.7 fold in H_2_O_2_ induced DLA cells [Fig 1A]. Increased absorbance of DCF with H_2_O_2_ was further confirmed through flow cytometry. Amount of ROS generation was indicated by shift in fluorescence [Fig 1B]. The result indicates that ROS accumulates in H_2_O_2_ induced DLA cells. Elevated level of ROS formation has been observed in animals suffering with different types of cancer including DL, which results in oxidative stress [43, 44].

### H_2_O_2_ modulates PI3K signaling

**Fig 2:**
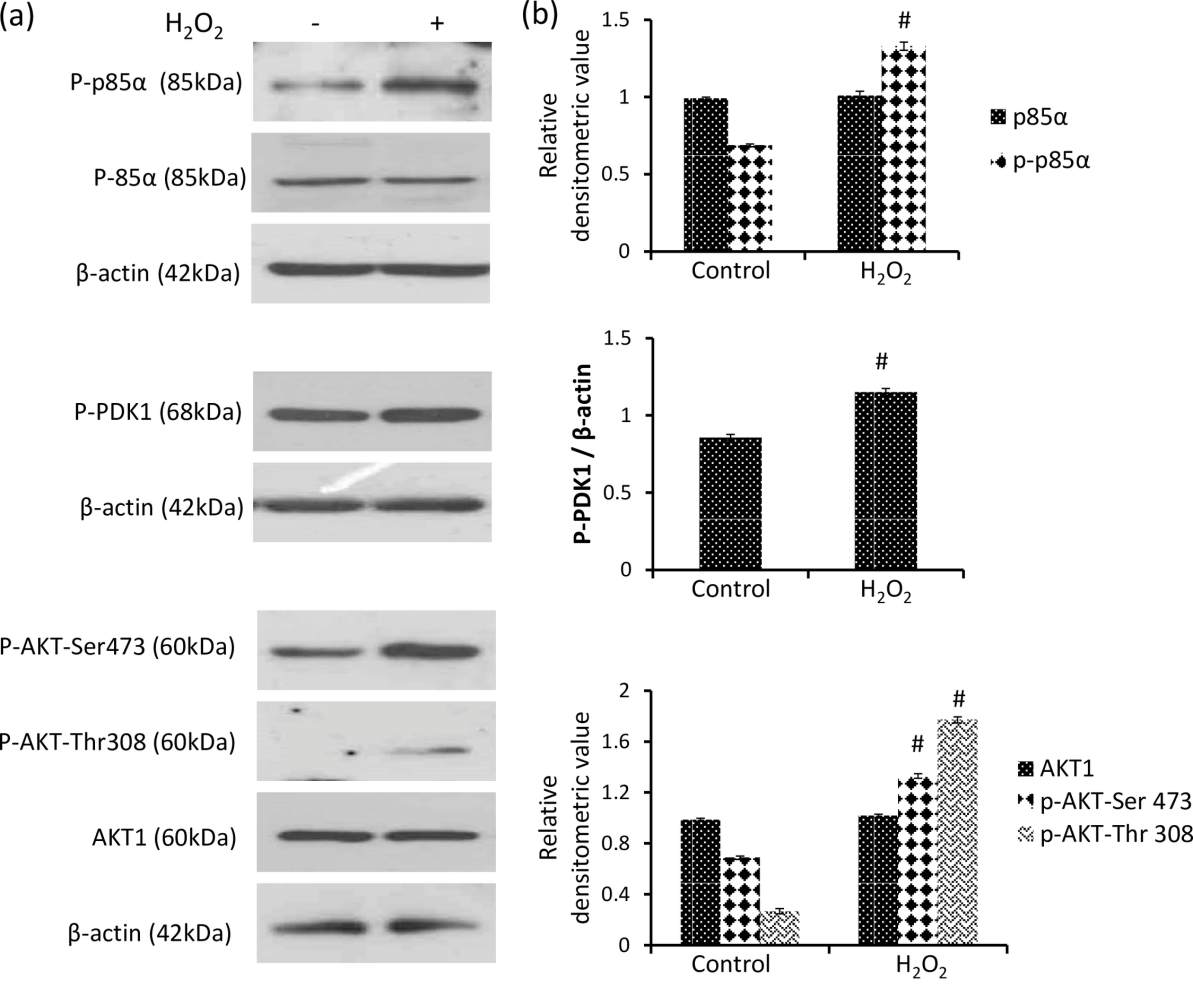
H_2_O_2_ activates PI3K signalling. (a) Western analysis of p85α, phospho p85α, phospho PDK1, phospho AKT Thr-308, phospho AKT Ser-473 and AKT1 (b) respective densitometric scanning of band after normalization with β-actin. Student’s t-test was used for statistical analysis. Data represent as mean ± S.E.M. of three independent experiments. * denotes significant differences at the level of p < 0.05 between H_2_O_2_-treated and control group.

PI3K signaling cascade has been linked to cell proliferation, survival and angiogenesis. Effect of H_2_O_2_ was investigated on the regulatory subunit p85α and downstream enzymes PDK1 and AKT. Phosphorylation of p85α was significantly increased by approximately 1.93 fold with H_2_O_2_, however protein level of p85α was found to be unchanged [Fig 2]. The result shows activation of PI3K by covalent modification without affecting its translation. Further, H_2_O_2_ increased phosphorylation of trafficking enzyme PDK1 significantly by approximately 34.4% in DLA cells [Fig 2].

AKT is an essential downstream target of PI3K signaling pathway that provides a survival signal. AKT activation is a multistep process involving both membrane translocation and phosphorylation. H_2_O_2_ increased phosphorylation of AKT at Ser-473 and Thr-308 significantly by approximately 93.4% and 6.63 fold respectively as compared to control DLA cells [Fig 2]. However, level of AKT1 was found unaffected showing that H_2_O_2_ modulates PI3K pathway by activation of AKT without affecting the protein level.

### H_2_O_2_ induces phosphorylation of BAD and ERK1/2 and the expression of TNFR1

**Fig 3:**
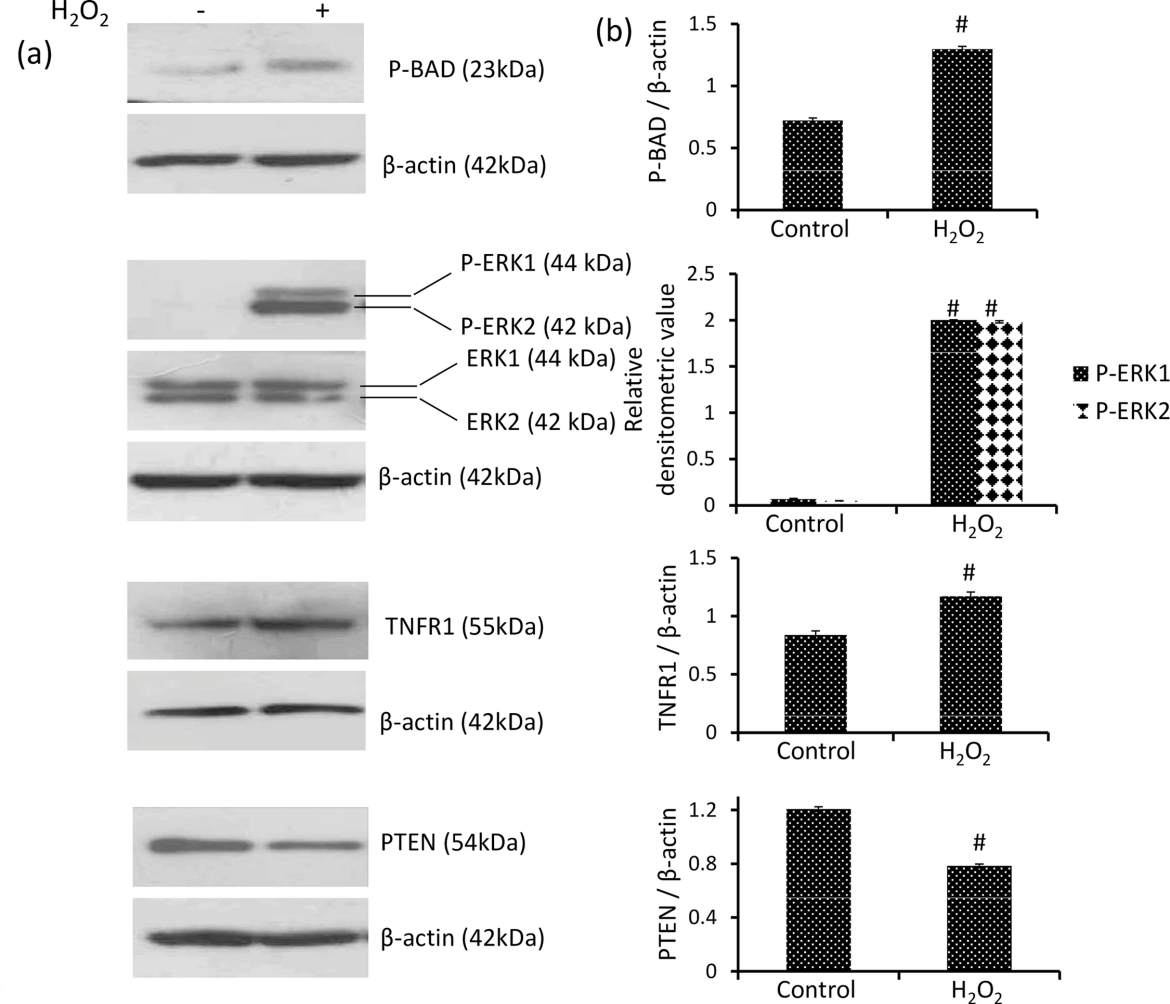
H_2_O_2_ induces the phosphorylation of BAD, ERK1/2 and TNFR1 level and down-regulates PTEN. (a) Western analysis of phospho BAD, ERK1/2,phospho ERK1/2, TNFR1 and PTEN (b) respective densitometric scanning of band after normalization with β-actin. Student’s t-test was used for statistical analysis. Data represent as mean ± S.E.M. of three independent experiments. * denotes significant differences at the level of p < 0.05 between H_2_O_2_-treated and control group.

BAD provides a link between cell death machinery and survival signaling. De-phosphorylated BAD forms heterodimers with Bcl-2 and Bcl-xL inactivating them and allowing Bax/Bak triggered apoptosis. BAD phosphorylation forms BAD-(14-3-3) protein heterodimers which leaves Bcl-2 free to inhibit Bax-triggered apoptosis. Therefore BAD phosphorylation is considered asanti-apoptotic, whereas dephosphorylation of BAD as pro-apoptotic signal. H_2_O_2_ increased phosphorylation of BAD significantly by approximately 80% in DLA cells as compared to control showing its inactivation [Fig 3].

The other kinase ERK1/2 is also known to inactivate BAD by phosphorylation. ERK1/2 is found to be activated in H_2_O_2_ induced DLA cells as indicated by its significant increase in phosphorylation as compared to negligible phosphorylation of ERK1/2 in control group [Fig 3]. Although, total level of ERK1/2 was unaffected by H_2_O_2_ exposure to DLA cells [Fig 3]_._

Further, the receptor TNFR1 is known to promote cancer growth via PI3K and NF-κB-dependent pathways. Level of TNFR1 was increased significantly by approximately 40% in H_2_O_2_ induced DLA cells as compared to control [Fig 3].

### H_2_O_2_ declines level of tumor suppressor PTEN

PTEN suppresses PI3K signaling by dephosphorylation of PIP3 to PIP2. The level of PTEN was observed to be decreased by approximately 35% in H_2_O_2_ induced DLA cells as compared to control [Fig 3].

### PI-103 and QUE decrease H_2_O_2_ induced ROS level

**Fig 4:**
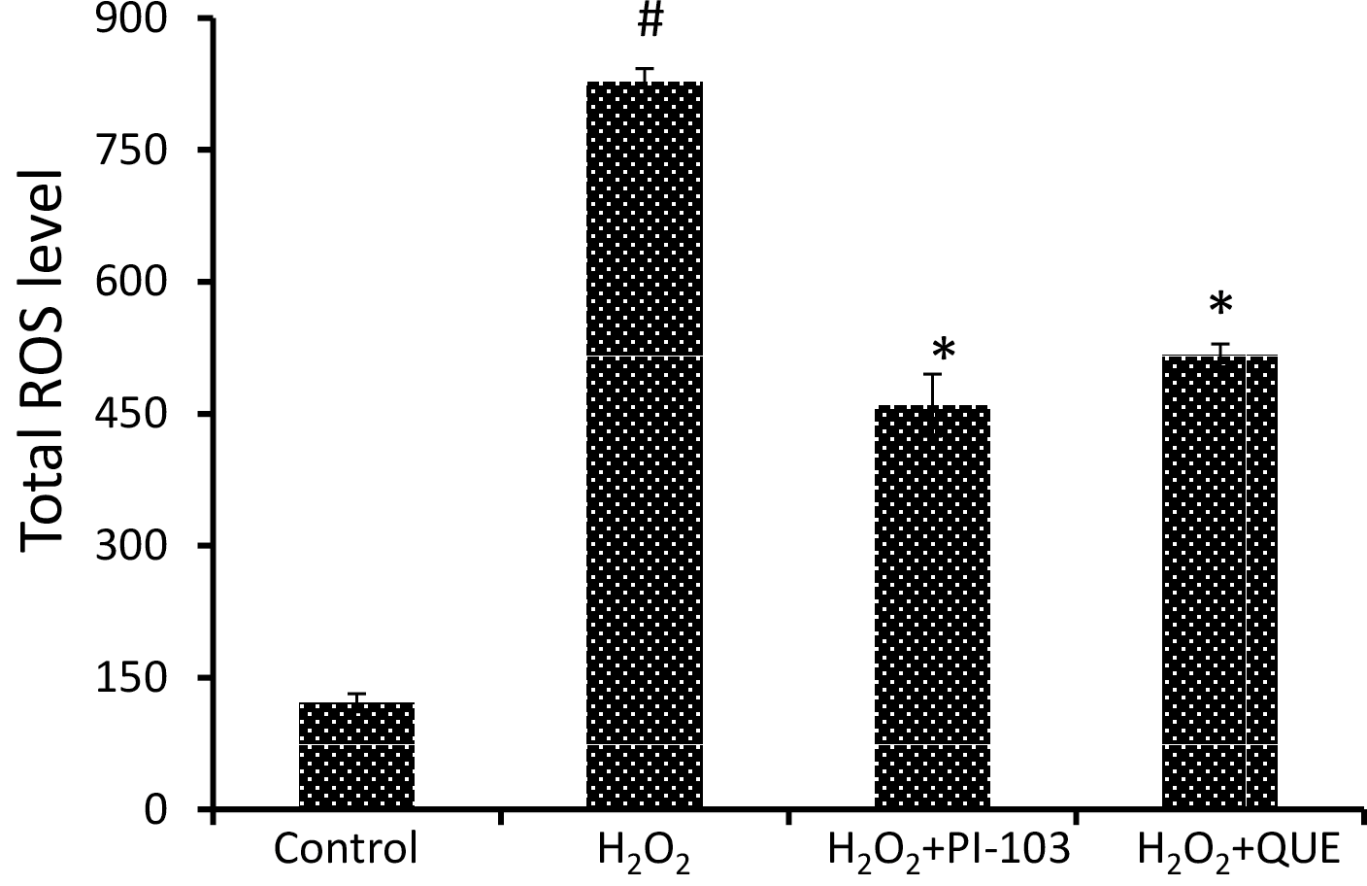
PI-103 and QUE decrease H_2_O_2_ induced ROS level. Homogenates of DLA cells from 3 set of each group were pooled separately and used for total ROS measurement. DLA cells were pre-treated with PI-103 and QUE; then post-treated with H_2_O_2_. Data represent as mean ± S.E.M. * (#) denotes significant differences at level of p < 0.05. # indicates significant difference between control *vs* H_2_O_2_-treated group; and * H_2_O_2_ treated groups *vs* PI-103/QUE treated group.

The effect of antioxidant QUE was compared with PI-103, a known inhibitor of PI3K on ROS level in terms of absorbance (fluorescence/mg protein) using green fluorescent dye, H_2_DCFDA. Both PI-103 and QUE decrease H_2_O_2_ induced ROS level approximately by 44% and 38% respectively in DLA cells [Fig 4].

### PI-103 and QUE suppresses cell proliferation

Effect of PI-103 and QUE on cell viability was analyzed in H_2_O_2_ induced DLA cells by MTT assay which measures mitochondrial dehydrogenase as cell viability. PI-103 and QUE decrease cell viability approximately by 47% and 41% respectively in H_2_O_2_ exposed DLA cells [S1 Fig]. However, H_2_O_2_ doesn’t significantly affect the DLA cell viability.

### PI-103 and QUE attenuate H_2_O_2_ induced phosphorylation of AKT

**Fig 5:**
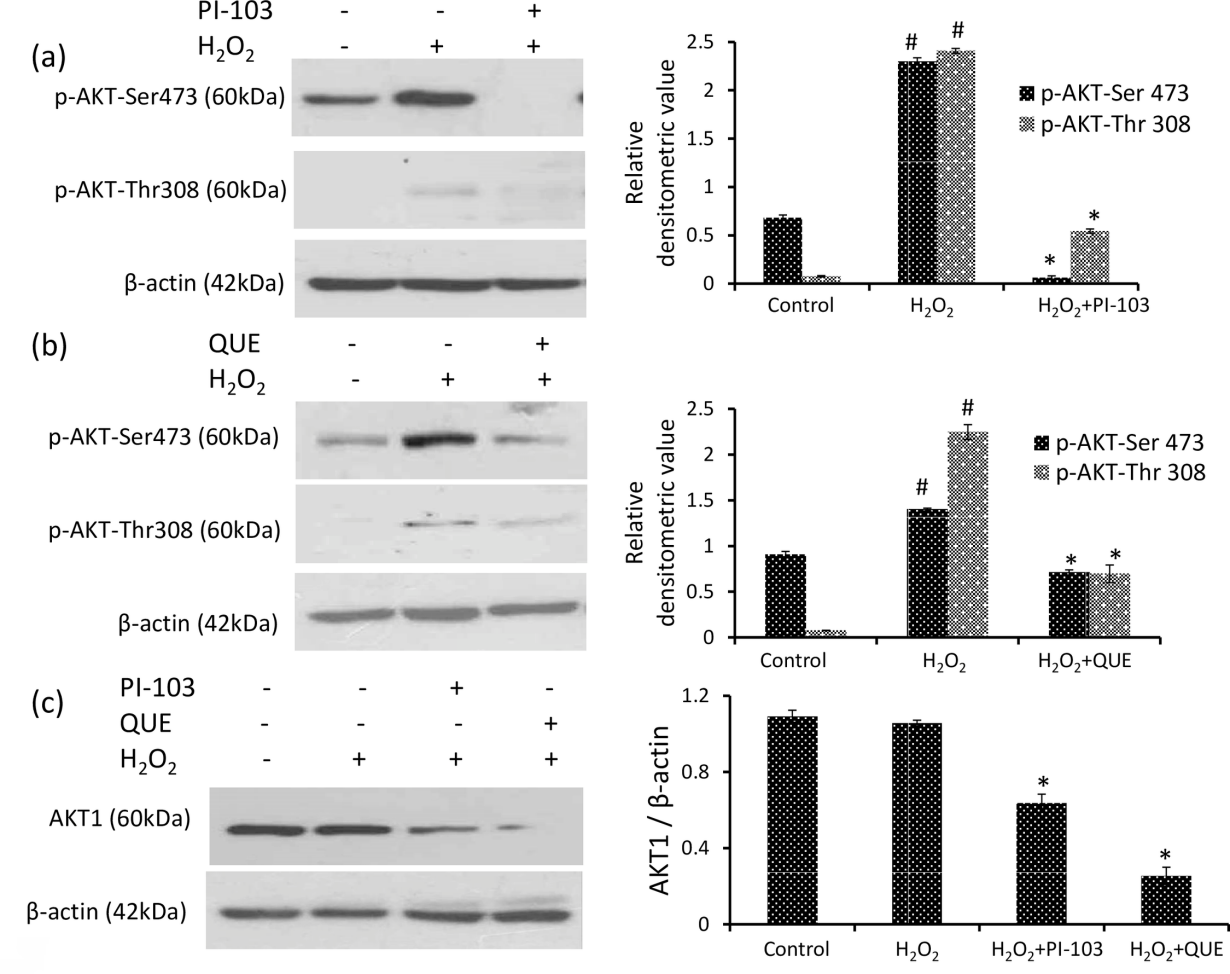
PI-103 and QUE attenuate phosphorylation of AKT Thr-308 & AKT Ser-473. (a) and (b) Western analysis of phospho AKT Thr-308, AKT Ser-473 and respective densitometric scanning of band after normalization with β-actin. (c) Western analysis of AKT1 and respective densitometric scanning of band after normalization with β-actin. DLA cells were pre-treated with PI-103 and QUE; then post-treated with H_2_O_2_. Data represent as mean ± S.E.M. * (#) denotes significant differences at level of p < 0.05. # indicates significant difference between control *vs* H_2_O_2_-treated group; and * H_2_O_2_ treated groups *vs* PI-103/QUE treated group.

Regulation of PI3K activity by QUE was compared with PI-103, an inhibitor of PI3K. PI-103 significantly decreased phosphorylation of AKT at Ser-473 and Thr-308 approximately by 97% and 77% respectively as compared to that of H_2_O_2_ induced DLA cells [Fig 5A]. QUE showed a similar effect as PI-103 showing significant decrease in phosphorylation of AKT at Ser-473 and Thr-308 approximately by 50% and 70% respectively as compared to H_2_O_2_ induced DLA cells [Fig 5B].

### PI-103 & QUE decline AKT1 level

Both PI-103 and QUE declined the protein level of AKT1 approximately by 40% and 75% respectively as compared to H_2_O_2_ induced DLA cells [Fig 5C]. QUE showed better effect than PI-103.

### PI-103 & QUE decrease H_2_O_2_ induced phosphorylation of PDK1

**Fig 6:**
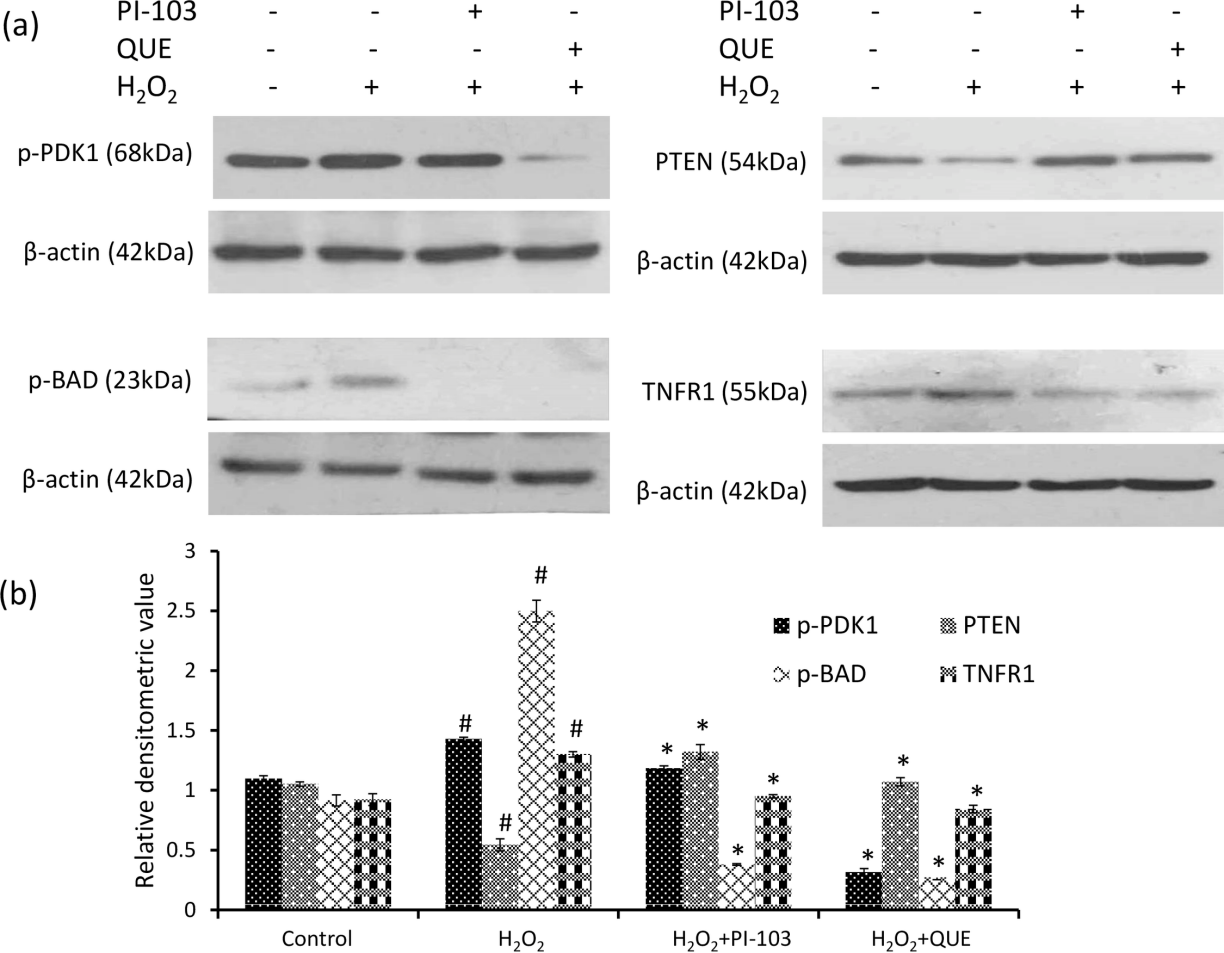
PI-103 and QUE attenuate H_2_O_2_ induced phosphorylation of PDK1, BAD, expression of TNFR1 and induce PTEN level. (a) Western analysis of phospho PDK1, PTEN, phospho BAD and TNFR1 (b) respective densitometric scanning of band after normalization with β-actin. DLA cells were pre-treated with PI-103 and QUE; then post-treated with H_2_O_2_. Data represent as mean ± S.E.M. * (#) denotes significant differences at level of p < 0.05. # indicates significant difference between control *vs* H_2_O_2_-treated groups; and * H_2_O_2_ treated groups *vs* PI-103/QUE treated group.

Both PI-103 and QUE decreased phosphorylation of PDK1 significantly by approximately 17% and 77% respectively as compared to H_2_O_2_ induced DLA cells [Fig 6]. QUE was found to be more efficient in regulation of PDK1 activity than PI-103.

### PI-103 & QUE attenuate H_2_O_2_ induced phosphorylation of BAD

BAD is downstream signaling protein of AKT. Phosphorylation of BAD is considered as anti-apoptotic, whereas its dephosphorylation favours pro-apoptotic activity. Both PI-103 and QUE significantly down-regulated phosphorylation of BAD approximately by 85% and 90% respectively as compared to H_2_O_2_ induced DLA cells reflecting dephosphorylation of BAD [Fig 6].

### PI-103 & QUE reduce H_2_O_2_ induced TNFR1 level

The result showed significant down-regulation in protein level of TNFR1 approximately by 27% and 35% by PI-103 and QUE respectively as compared with H_2_O_2_ induced DLA cells [Fig 6] indicating reduced cell survival.

### PI-103 & QUE induce PTEN level

The level of tumor suppressor PTEN, a negative regulator of PI3K is increased significantly by both PI-103 and QUE approximately by 2.43 fold and 2 fold respectively as compared to H_2_O_2_ induced DLA cells [Fig 6]. Induced level of PTEN reflects down-regulation of PI3K activity by PI-103 as well as QUE.

### PI-103 & QUE down-regulate PKCα level

**Fig 7:**
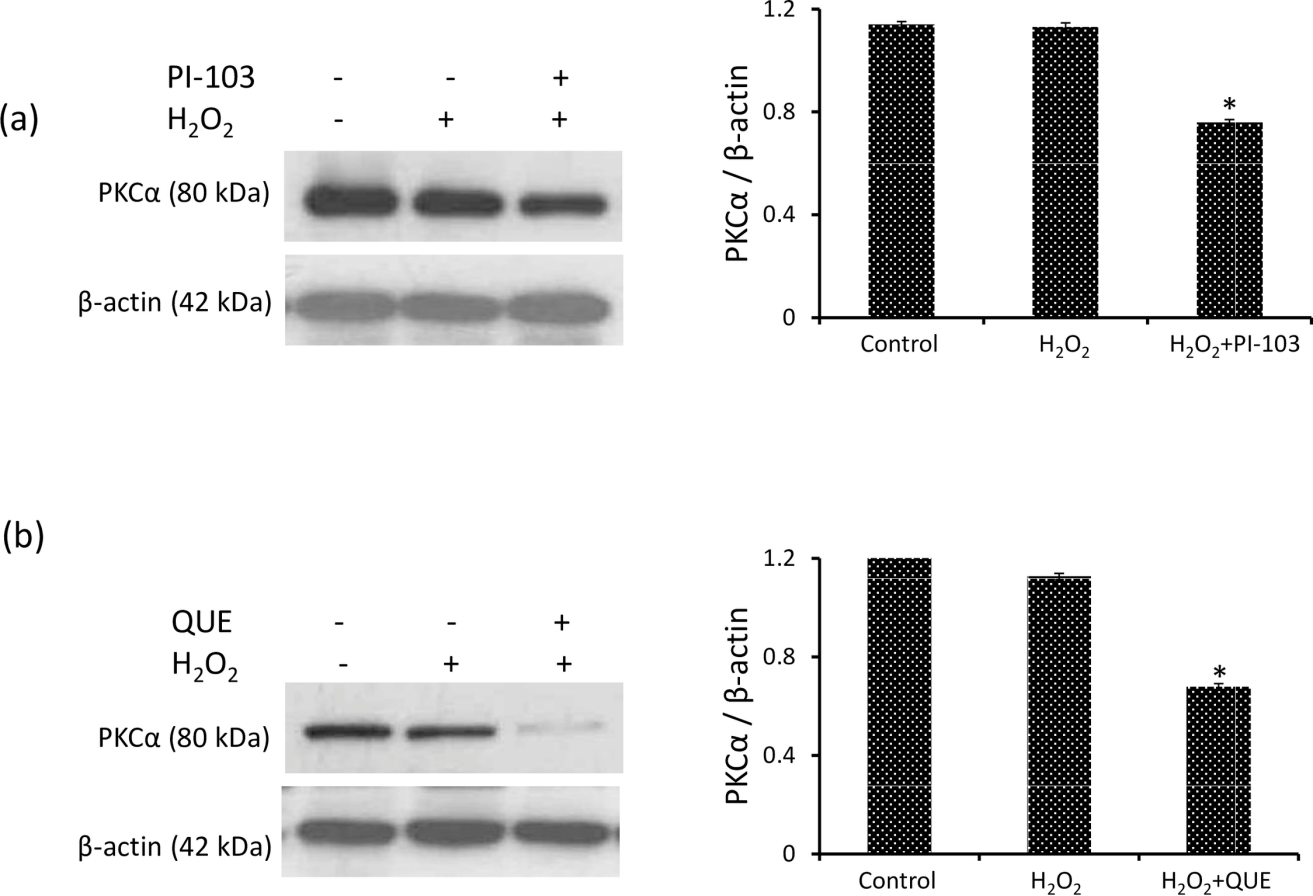
PI-103 & QUE downregulate PKCα level. (a) and (b) Western analysis of PKCα and respective densitometric scanning of band after normalization with β-actin. DLA cells were pre-treated with PI-103 and QUE; then post-treated with H_2_O_2_. Data represent as mean ± S.E.M. * (#) denotes significant differences at level of p < 0.05. # indicates significant difference between control *vs* H_2_O_2_-treated groups; and * H_2_O_2_ treated groups *vs* PI-103/QUE treated group.

PDK1 regulates the activity of downstream protein PKCα, a key regulator of cell growth and differentiation in mammalian cells and activation of PKCα is believed to promote tumor progression. Level of PKCα was unaffected by H_2_O_2_ exposure to DLA cells. However, both PI-103 and QUE attenuate the level of PKCα approximately by 32% and 42% respectively as compared to H_2_O_2_ induced DLA cells [Fig 7A and 7B]. The effect of QUE was better than that of PI-103.

The overall result shows similar effect of QUE and PI-103, which supports that antioxidant QUE is a potential down-regulator of PI3K-AKT signaling pathway.

## Discussion

Previously we have reported hyper-activation of PI3K-AKT pathway involved in tumor cell survival in Dalton’s lymphoma bearing mice and QUE attenuates the hyper-activation bringing the pathway towards normal [11]. The molecular mechanism of PI3K-AKT pathway has been established by using PI-103, a potent and selective inhibitor of PI3K [41]. PI-103 is reported to show therapeutic activity against a range of cancer by inhibition of angiogenesis, invasion and metastasis [42, 46]. It has a number of metabolic hotspots, especially the phenol ring, which are shown to be extensively glucuronidated, resulting in rapid plasma and tissue clearance [41]. In the present study the regulation of PI3K-AKT pathway by PI-103 is compared with QUE in lymphoma cells *in vitro*. QUE is a dietary antioxidant with almost no side effect as reported by Harwood *et al*., 2007. The lymphoma cells were exposed to oxidative insult *in vitro* by 1mM H_2_O_2_ for 30 min.

Oxidative stress is an evident feature of almost all cancerous cells as compared to normal, which activates several signaling cascade to achieve adaptation for survival and immortalization [47–49]. Numerous signaling pathways are involved in tumor cell survival including PI3K-AKT pathway [1, 4]. Hyper-activation of PI3K pathway is frequent in human cancer and has been considered as a major drug target in cancer treatment [5–9]. PI3K is activated by several factors including oxidative stress. Previously we have reported that QUE attenuates ROS accumulation in DL mice as well as in HepG2 cells [11, 12].

In present study, short time exposure of H_2_O_2_ to ascite cells from DL mice *in vitro* is found to induce accumulation of ROS causing oxidative stress. The ROS level was decreased after treatment of H_2_O_2_ induced DLA cells with antioxidant QUE as well as PI3K inhibitor PI-103. ROS is involved in various cellular processes that positively and negatively regulate cell fate. It is reported to promote cell proliferation and survival in several cell types [29]. On the other hand, it has been shown to induce apoptosis in certain cell types and organs [37, 50]. Oxidative stress is suggested to be a key pathogenic factor in cisplatin-induced ototoxicity *in vivo* [51].

The effect of oxidative stress is analyzed on signaling pathway of proto-oncogene PI3K. The best elucidated mechanism of PI3K activation involves association of p85α subunit with specific phospho-tyrosine on membrane. Under basal condition, regulatory subunit p85α stabilizes catalytic subunit p110α and inhibits its catalytic activity. Phosphorylation of p85α alleviates the inhibition, causing activation of PI3K [15]. Increased phosphorylation of p85α has been linked with various cancers [10, 13, 15]. H_2_O_2_ is found to increase phosphorylation of p85α in DLA cells *in vitro* without affecting its level. The result reflects that H_2_O_2_ activates PI3K by increasing tyrosine kinase activity [52]. Earlier we have reported down-regulation of PI3K by antioxidant QUE in ascite cells of DL mice [11]. PI3K catalyzes phosphorylation of lipid associated PIP2 in to PIP3, which recruits AKT as well as PDK to plasma membrane. PDK dependant phosphorylation of AKT leads to its activation [16].

PDK1 is a crucial kinase required for normal mammalian development [19, 20]. However, it is frequently elevated in cancer with parallel increased phosphorylation of downstream kinase AKT at Thr-308 [20]. Exposure of lymphoma cells to H_2_O_2_ to DLA cells increased phosphorylation of PDK1. Both QUE and PI-103 show down-regulation of phosphorylation of PDK1 in H_2_O_2_ induced DLA cells.

Full activity of AKT requires phosphorylation at Ser-473 and Thr-308. Since hyperactivation of AKT supports tumor cell survival, it becomes strategic target in cancer therapeutics [16, 53]. PDK1 is responsible for phosphorylation of Thr-308 [17, 18]. Our results demonstrate that H_2_O_2_ increased phosphorylation of both Ser-473 and Thr-308 region of AKT without affecting its level in DLA cells. The result suggests that H_2_O_2_ activates PI3K signaling pathway via PDK1 and AKT. An inhibitory effect on H_2_O_2_ mediated AKT activation is shown by PI-103 as well as QUE. In addition, the level of AKT1 is also decreased by both PI-103 and QUE where QUE shows better effect than PI-103. A similar decrease in AKT1 level after QUE treatment to DL mice has been reported earlier [11]. QUE has classically been defined as a broad spectrum inhibitor against PI3K [54]. QUE inhibits AKT1/2 on platelet spreading [54]. Inhibition of AKT1/2 by QUE suggested that the flavonol either by a direct effect on this Ser/Thr kinase or most significantly as a regulation of the enzyme downstream of PI3K. PI-103 has been reported to inhibit PI3K/AKT activity and blocks the proliferation in human leukemic cell lines [55].

This is consistent with our finding of declined level of tumor suppressor PTEN in DLA cells exposed to H_2_O_2_. Tumor suppressor PTEN exerts feedback regulation of PI3K signaling by dephosphorylation of PIP3 and thus imposes negative regulation on AKT activity. PTEN is frequently lost or mutated in cancer [56, 57]. Our finding is supported by earlier report of PTEN inactivation by H_2_O_2_ [58]. Modulation in activity of both AKT and PTEN play a central role in tumorigenesis. Upregulation of tumor suppressor PTEN level by QUE is similar to the effect of PI-103 in DLA cells. A similar increase in tumor suppressor p53 level by QUE has been reported earlier in DL mice [11]. Upregulation of PTEN by QUE and PI-103 indicates down-regulation of AKT signaling.

AKT promotes cell survival by phosphorylating and inhibiting pro-apoptotic protein BAD [22]. BAD is a member of Bcl-2 family which promotes cell death by displacing Bax from binding to Bcl-2 and Bcl-xL [23, 59–61]. BAD function is regulated by phosphorylation. Dephosphorylated BAD forms a heterodimer with Bcl-2 and Bcl-xL, inactivating them and allowing Bax/Bak triggered apoptosis. Phosphorylation of BAD leads to its binding to 14-3-3 protein, which leaves Bcl-2 free to inhibit Bax-triggered apoptosis. Thus BAD phosphorylation promotes cell survival. H_2_O_2_ increases phosphorylation of BAD in DLA cells. The result is supported by earlier report [34]. Both QUE and PI-103 decreased phosphorylation of BAD in H_2_O_2_ induced cells suggesting reduced survival of DLA cells. Our results are in accordance with the report that AKT phosphorylates BAD to promote cell survival *in vivo* and *in vitro* [22]. Inactivation of BAD is also mediated through phosphorylation by ERK. Activation of ERK1/2 inhibits apoptosis by inactivation of caspase 8. PI3K activation is responsible for ERK1/2 phosphorylation [25]. Our result shows increased phosphorylation of ERK1/2 without affecting its level in H_2_O_2_ induced DLA cells.

PKCα is another trafficking molecule of PDK1, which is a key regulator of cell growth and differentiation and its activation is believed to promote tumor progression [62]. Level of PKCα is down-regulated by both QUE and PI-103 in H_2_O_2_ induced DLA cells. The result is in consistence with our previous reports in DL mice and HepG2 cells [11, 12].

Abundance of TNFR1 is known to promote cell survival via PI3K and NF-κB dependant pathway [58, 63–68]. Increased level of TNFR1 by H_2_O_2_ exposure supports induced survival of DLA cell. The result correlates with increased TNFR1 expression after exposure of ROS in neuronal cells [69]. Further, both QUE and PI-103 down-regulate level of TNFR1. Reduced level of TNFR1 by QUE is in accordance with our previous report in DL mice [35]. Together, down-regulated BAD and TNFR1 by QUE suggests reduced survival of H_2_O_2_ induced DLA cells.

**Fig 8:**
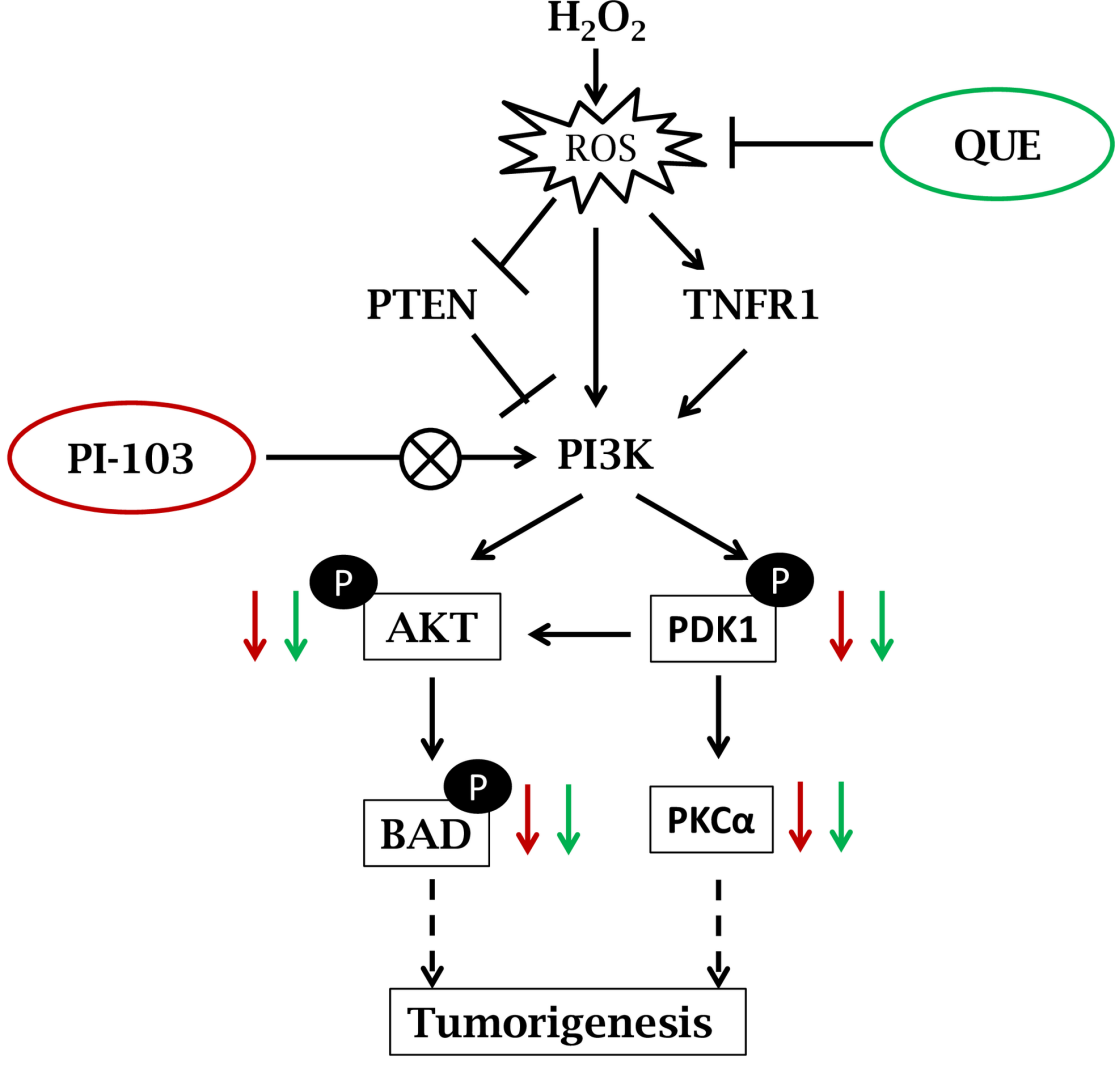
Schematic representation showing PI-103 and QUE mediated modulation of PI3K-AKT signaling pathway. Exposure of H_2_O_2_ induces ROS accumulation, which in turn activates TNFR1 and PI3K level. Activated PI3K phosphorylates AKT via activated PDK1 and thereby induces downstream survival factor BAD. This is consistent with reduced level of tumor suppressor PTEN—negative regulator of PI3K pathway. Enhanced level of ROS by H_2_O_2_ is suppressed by both PI-103 & QUE which in turn down-regulates phosphorylation of AKT, PDK1 and BAD. Additionally, QUE and PI-103 increase the level of PTEN and decrease TNFR1 as well as PKCα.

The results of current study suggest that H_2_O_2_ activates PI3K-AKT signaling via increasing ROS level. Overall effect of QUE is found similar to that of PI-103. Both PI-103 & QUE reduce phosphorylation of AKT and PDK1 whereas up-regulate level of tumor suppressor PTEN. This is consistent with decreased level of cell survival factors; p-BAD and TNFR1 in DLA cells *in vitro*. PI-103 and QUE mediated modulation of PI3K-AKT signaling pathway in DLA cells exposed to H_2_O_2_ is represented by schematic diagram [Fig 8]. The findings suggest that QUE modulates PI3K-AKT signaling pathway suppressing survival signals in prevention of lymphoma growth. This study may provide the base for implication of PI3K-AKT signaling in cancer targeting.

## Supporting Information

S1 Fig PI-103 and QUE attenuate cell proliferation. DLA cells were pre-treated with PI-103/QUE and post-treated with H_2_O_2_, then subjected to MTT dye reduction assay. The percentage viable cells (relative to control) were plotted against concentration. The data at each point represent mean ± S.E.M. * denotes significant differences at level of p < 0.05 between H_2_O_2_ treated group vs PI-103/QUE-treated groups.
(TIF)
