# Supplementary figures and images for: PI-103 and Quercetin Attenuate PI3K-AKT Signaling Pathway in T- Cell Lymphoma Exposed to Hydrogen Peroxide
Source: PLoS One. 2016 Aug 5;11(8):e0160686. doi: 10.1371/journal.pone.0160686 (PMC4975451; doi:10.1371/journal.pone.0160686)

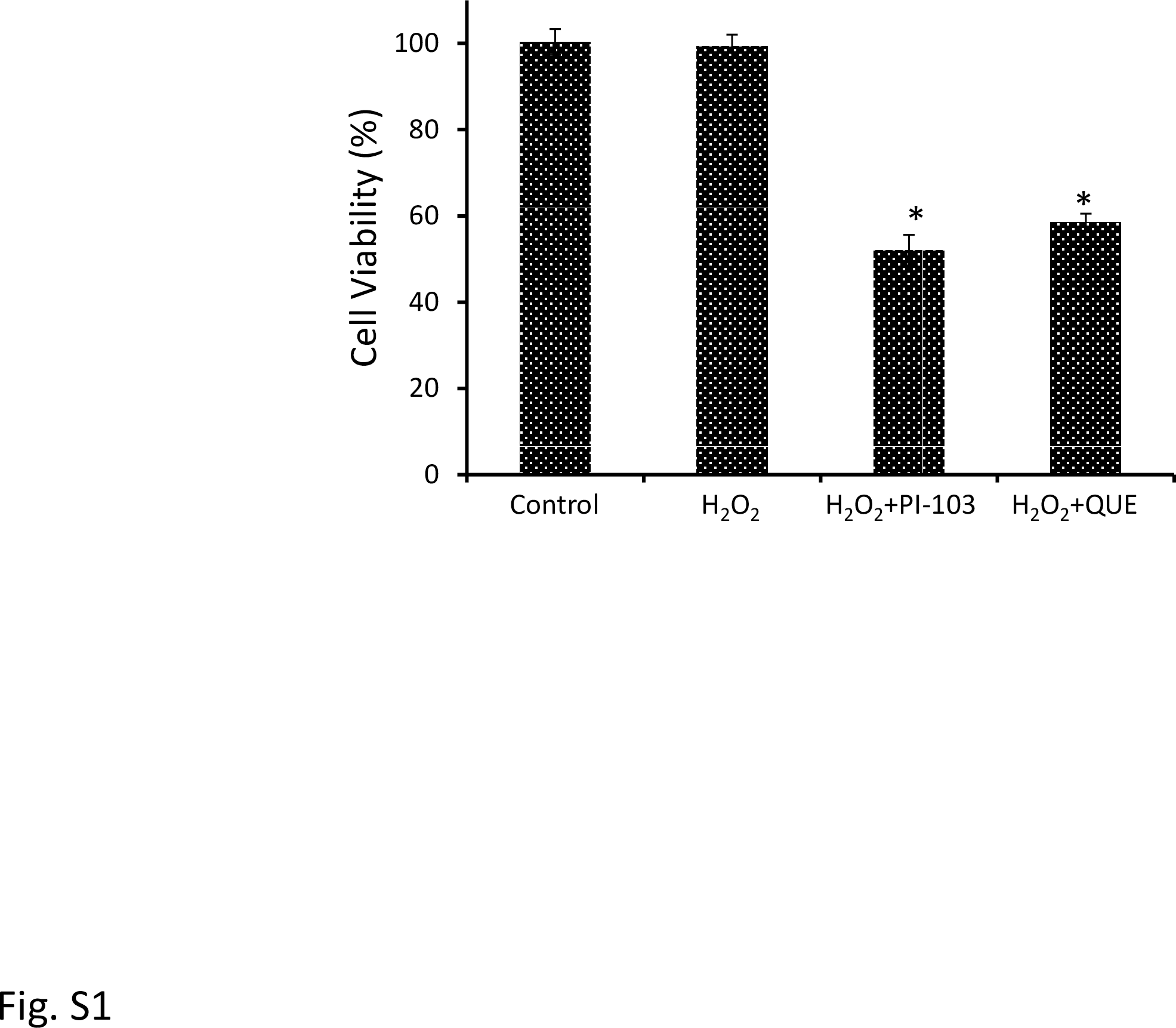

Supplement: S1 Fig — DLA cells were pre-treated with PI-103/QUE and post-treated with H2O2, then subjected to MTT dye reduction assay. The percentage viable cells (relative to control) were plotted against concentration. The data at each point represent mean ± S.E.M. * denotes significant differences at level of p < 0.05 between H2O2 treated group vs PI-103/QUE-treated groups. (TIF) [file pone.0160686.s001.tif]
